# Modeling the grid cell activity based on cognitive space transformation

**DOI:** 10.1007/s11571-023-09972-w

**Published:** 2023-04-20

**Authors:** Zhihui Zhang, Fengzhen Tang, Yiping Li, Xisheng Feng

**Affiliations:** 1 University of Science and Technology of China, Hefei, 230026 China; 2 State Key Laboratory of Robotics, Shenyang Institute of Automation, Chinese Academy of Sciences, Shenyang, 110016 China; 3 Institutes for Robotics and Intelligent Manufacturing, Chinese Academy of Sciences, Shenyang, 110016 China

**Keywords:** Grid cell, Place cell, Spatial transformation, Neural computational model, Cognitive space

## Abstract

The grid cells in the medial entorhinal cortex are widely recognized as a critical component of spatial cognition within the entorhinal-hippocampal neuronal circuits. To account for the hexagonal patterns, several computational models have been proposed. However, there is still considerable debate regarding the interaction between grid cells and place cells. In response, we have developed a novel grid-cell computational model based on cognitive space transformation, which established a theoretical framework of the interaction between place cells and grid cells for encoding and transforming positions between the local frame and global frame. Our model not only can generate the firing patterns of the grid cells but also reproduces the biological experiment results about the grid-cell global representation of connected environments and supports the conjecture about the underlying reason. Moreover, our model provides new insights into how grid cells and place cells integrate external and self-motion cues.

## Introduction

With the discovery of grid cells in the medial entorhinal cortex (mEC), the study of the circuit of spatial representation has evolved in stage (Moser et al. 2017). Grid cells have hexagonally arranged firing fields that almost cover the entire space of the environment that a freely moving rodent has passed through (Giocomo et al. 2011; Wagatsuma and Yamaguchi 2007). Grid cells are initially discovered in mEC of rat (Hafting et al. 2005). In addition, the grid cells also are found in mice, bats, monkeys, and humans (Rowland et al. 2016). So researchers think the grid cells play an important role in spatial representation (Yan et al. 2016; Zeng and Si 2021). To explore the secret of grid cells, subsequent researches focus on the property of grid cells. The firing fields of grid cells have three dimensions including spacing, orientation, and phase (Guanella et al. 2007). Different grid cells have different spacing, orientations and phases. But neighboring cells maintain the relative relationship of orientation and phase. This shows that gird cells may provide some odometry information for place cells (McNaughton et al. 2006) in the hippocampus. Another property of grid cells is that the firing fields remain a steady hexagonal pattern although they receive various head directions from head direction cells (Taube et al. 1990) and speed information from speed cells (Kropff et al. 2015). As for the spacing, it has been witnessed that grid cells that have increasing spacing are arranged along with the dorsoventral axis of mEC. Moreover, the grid cells in a single module have the same spacing and orientation but different phases (Stensola et al. 2012). As the research progress, the grid cells generally are regarded as a space metric or a module of path integration in cognitive map (Jacob et al. 2019). Simultaneously, many models of grid cells were proposed to explain these biological phenomena and explore the latent mechanism. Current most-important computational models of grid cells can be roughly divided into three classes, including oscillatory interference(OI) models (Burgess et al. 2007; Pastoll et al. 2013) continuous attractor network(CAN) models (Burak and Fiete 2009; Fuhs 2006; Guanella et al. 2007), and single-cell adaptation models (Kropff and Treves 2008; Monsalve-Mercado and Leibold 2017; D’Albis and Kempter 2017).

The CAN models have been successfully used in many cognitive neurons such as head direction cells and place cells (Zhang 1996; Fuhs 2006; McNaughton et al. 2006; Guanella et al. 2007). In CAN models, the neurons are connected by different weights according to their distance in the network. In the CAN models of place cells, the networks are stimulated by external inputs and can drive the bump moving by internal self-motion cues (Burak and Fiete 2009; Guanella et al. 2007; Shipston-Sharman et al. 2016). This can be similar to the path integration process of animals in navigation. In the case of grid cells, the CAN networks are stimulated by the functions of Mexican-hat connectivity or Lincoin-hat connectivity to acquire the hexagonal firing fields. This make CAN models of grid cell remain hexagonal firing bumps (Burak and Fiete 2009).

Another main models of grid cells are OI models. They are originally proposed to explain the theta phase procession in place cells (Baker and Olds 2007; OKeefe 1976). In those models, the grid-like patterns can be generated by interference between different oscillators which are derived from the velocity of animal movement (D’Albis and Kempter 2017; Bush and Schmidt-Hieber 2018). In original OI models, a baseline frequency is assumed to exist in mEC and remain a constant as an oscillator (Moser et al. 2017). its frequency generally is about 5–12 Hz in rodents. Other oscillators are controlled by the speed of the movement. So those oscillators are called velocity-controlled oscillators(VCO) (D’Albis and Kempter 2017). Then the VCO and baseline oscillator performs an interference to generate the grid-like patterns.

Single-cell plasticity models are another grid-cell models that emphasize the roles of external cues and learning (Kropff and Treves 2008). The classic single-cell plasticity model (Kropff and Treves 2008) suggests that grid-like patterns may originate from a competition of grid-cell neurons. The competition is controlled by spatially selective synaptic inputs and spike-rate adaptation (Peron and Gabbiani 2009). The inputs of external can be stretched by Hebbian synaptic plasticity to affect the output activity of grid cells. The spacing of grid cells is determined by the time constant of adaptation and the average running speed of the animal.

As mentioned above, several main models of grid cells have been proposed to understand the latent mechanism of the grid-like firing patterns of grid cells. And most models can generate the grid pattern and try to reveal the latent mechanism of grid cells. However, there still is a mysterious area about the interaction between place cells and grid cells. Easily evidence observed that the grid pattern remained stable in the dark (Hafting et al. 2005). It demonstrates that the grid cells can utilize the self-motion cues to perform the path integration. Recent research shows that grid cells may need external cues to adjust the firing patterns. Chen et al. observed that the absence of external input results in the disruption of grid cell firing in the mouse (Chen et al. 2016). In addition, environmental shape and in particular enclosure boundaries can change the firing patterns of grid cells (Krupic et al. 2015, 2016). This evidence demonstrates the grid cells need both external cues and self-motion inputs. However, the OI models more focus on the self-motion inputs and the single-cell adaptation models emphasize the external cues. Although the CAN models can utilize both self-motion inputs and external cues, the external cues make little contribution to the generation of grid-like patterns. As the CAN models can generate grid-like patterns only in the presence of self-motion inputs. The external cues are only utilized to correct the errors from the self-motion inputs. Different from CAN models, our idea is to tightly couple the self-motion cues and external cues to provide an insight to understand how place cells and grid cells interact with each other.

In this paper, we propose a new model to provide a new insight to understand how the place cells and grid cells integrate external and self-motion cues. we are inspired by the topic of egocentric and allocentric representations of space in rodent brains (Wang et al. 2020) and think that the reason for grid-like patterns comes from the projection of grid-cell cognitive space in the physical world. The cognitive map have been proposed by Tolman in 1948 early (Tolman 1948). It is thought to be the representation of the physical environment in cognitive space. In our model, the cognitive space contains place-cell and grid-cell cognitive space. The inputs from the medial entorhinal cortex (MEC) are transformed from place-cell cognitive space into grid-cell cognitive space, which is referred to as cognitive space transformation. our model utilizes the external cues by place cells, which is consistent with the evidence that place cells mainly receive the visual cues (Zhong and Wang 2021). The external cues stimulate different place cells to provide different local frames in place-cell cognitive space. Based on these place-cell local frames, the self-motion inputs are used to perform path integration and are transformed into grid-cell cognitive space. In this way, the ideal grid-like firing patterns are produced. Our model proposes a theoretical framework of the interaction between place cells and grid cells for encoding and transforming positions between the local frame and global frame. And our model is laterally supported by the experiment about grid-cell global representation in connected environment (Carpenter et al. 2015). The results of our model reproduce the experiment phenomena and predict the results in a novel connected environment.

For clarity, the novel contributions of our work are listed as follows:

- We propose a theoretical framework of the interaction between place cells and grid cells for encoding and transforming positions between the local frame and global frame.
- Our model provides an insight to understand how the place cells and grid cells integrate external and self-motion cues.
- Our model reproduces biological experiment (Carpenter et al. 2015) about the grid-cell global representation of connected environments and predicts grid-cell activity in a novel connected environment.

## Results

### Model description

**Fig. 1:**
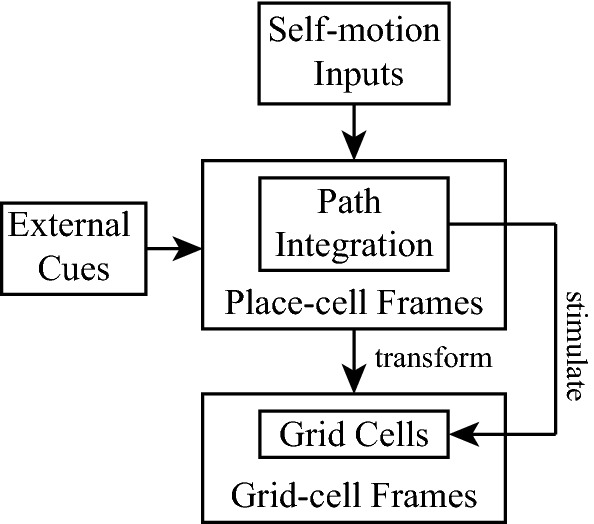
The overall framework of the proposed model. The external cues in the model provide different local frames referred to as place-cell frames. The place-cell frames provide a basis for self-motion inputs to integrate. The integrated self-motion inputs are transformed into grid-cell cognitive space via these place-cell frames

In this paper, we propose a new grid cell model based on cognitive spatial transformation and provide an insight to understand how the place cells and grid cells integrate external and self-motion cues. Much evidence have shown that grid cells are affected by external cues (Chen et al. 2016; Krupic et al. 2015, 2016; Stensola et al. 2015; Monsalve-Mercado and Leibold 2020; Pérez-Escobar et al. 2016). Specifically, the grid cells have local representation and global representation for the environment (Carpenter et al. 2015; Wang et al. 2020; Wernle et al. 2018). Furthermore, the local representation is determined by external cues of the environment where animals move. The activities of place cells are determined by external cues (O’Keefe and Burgess 1996; O’Keefe and Conway 1978). The papers (Fyhn et al. 2004; Bonnevie et al. 2013) reveal that place cells affect the activities of grid cells. So we naturally assume that the local representation of grid cells may derive from place cells via external cues. In addition, some evidence has suggested that the putative functional magnetic resonance imaging marker is also found when animals are stimulated along two abstract dimensions (Whittington et al. 2022). So the external cues are assumed to provide such two abstract dimensions marked as a local frame to represent the current environment by stimulating the place cells. We naturally use local frames to represent the place-cell cognitive space. As shown in Fig. 1, the external cues in the model provide different local frames referred to as place-cell frames. The place-cell frames provide a basis for self-motion inputs to integrate. The integrated self-motion inputs are transformed into grid-cell cognitive space via these place-cell frames. In the grid-cell cognitive space, every grid cell has its basis vectors that can be constructed via the preferred spacing, orientation and phase of the grid cell. For simplicity, the cognitive space described by the basis vectors of the grid cell is referred to as the grid-cell frame. Our model makes grid cells acquire the local representation due to various external cues. During the pipeline, there are two key steps for our model to generate grid patterns. One is the transformation from the place-cell frames to the grid-cell cognitive space. The other is the construction of the basis vectors of the grid-cell cognitive space, including the selection of spacing, orientation, and phases.

**Fig. 2:**
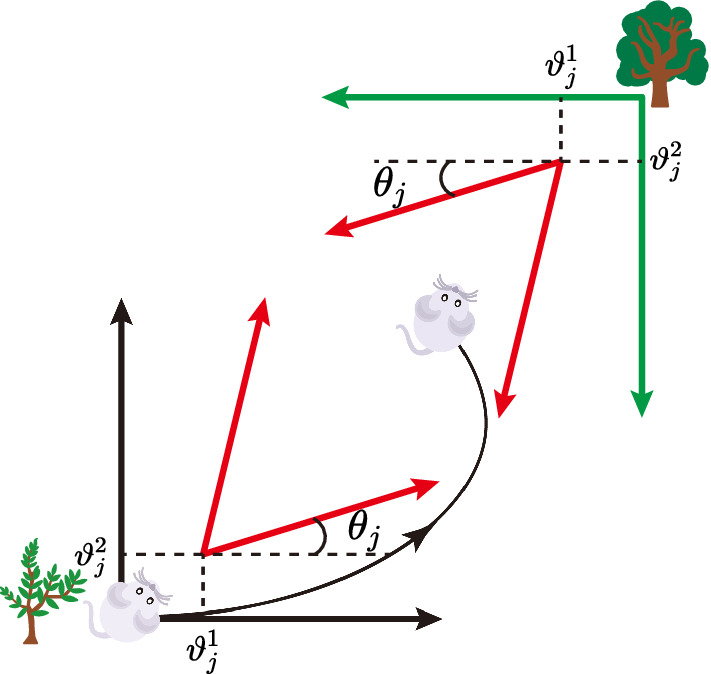
Different frames in cognitive space. In the initial phase, the rat begins to move and the initial place-cell frame $\mathcal {C}_1$ plotted by the black color is determined by the external cues, i.e., the left-bottom tree. The grid-cell frame is built based on the place-cell frame $\mathcal {C}_1$. The preferred orientation and phases of the grid cell represent the relative position relationship in cognitive space. Then self-motion inputs of the rat are projected into the cognitive space via the grid-cell frame. When the rat moves a distance, the external cues change in the current environment, which is depicted by another tree in the right-top corner. The new external cues enable stimulate the place cell to acquire a new place-cell local frame $\mathcal {C}_2$. Although the relationship between the place-cell frame and grid-cell frame remains unchanged, the grid firing field will be altered relative to the global frame, i.e., $\mathcal {C}_1$ due to different place-cell frames

As shown in Fig. 2, a rat is moving in the environment. Given that the starting point $\varvec{P^{p}_i}=(x^{p}_i,y^{p}_i)$, the position of the next moment $\varvec{P^{p}_{i+1}}=(x^{p}_{i+1},y^{p}_{i+1})$ in the initial local frame $\mathcal {C}$ can be acquired by the velocity $\varvec{v} (t)$ and angular $\alpha (t)$ from speed cells and head direction cells.

1 $$\begin{aligned} \varvec{P^p_{i+1}} =\varvec{P^p_i}+ \Delta \varvec{P^p_{i}} \end{aligned}$$

where $\Delta \varvec{P^p_{i}} = (\Delta x^p_i,\Delta y^p_i)$ collects the movements in the initial local frame $\mathcal {C}$.

2 $$\begin{aligned} \Delta x^p_i= & {} \int _{t_{i}}^{t_{i+1}}\varvec{v}(t) \cdot cos\alpha (t) \ \text {d}t \end{aligned}$$

3 $$\begin{aligned} \Delta y^p_i= & {} \int _{t_{i}}^{t_{i+1}}\varvec{v}(t) \cdot sin\alpha (t) \ \text {d}t \end{aligned}$$

The initial local frame is determined by external cues in the current environment observed by the rat. When the rat moves in $\mathcal {C}$, its positions in the current environment are transformed into grid-cell cognitive space. In this space, a grid cell has its basis vectors to represent the inputs of positions. The basis vectors are constructed by preferred spacing, orientation and phases of the grid cell. To better describe our model, a grid cell can be defined as a vector as follows:

4 $$\begin{aligned} \varvec{G_j} = [s_j,\theta _j,\vartheta _j^1,\vartheta _j^2], j\in \varvec{Z^+} \end{aligned}$$

where $\varvec{G_j}$ is the *j*th grid cell vector, $s_j \in R^{+}$ is the spacing of the grid cell $\varvec{G_j}$, $\theta _j \in [0,\pi /3]$ is the orientation of the grid cell $\varvec{G_j}$, and $\varvec{\vartheta _j}=[\vartheta _j^1,\vartheta _j^2],\vartheta _j^k\in [0,2\pi ],k=1,2$ are the phases of the grid cell $\varvec{G_j}$. The firing field of a grid cell can cover the explored physical environment (Bonnevie et al. 2013; Bush et al. 2015). In other words, any position in the environment can map to a value in the grid-cell cognitive space. By this mapping, a position in the place-cell frame can be connected with the elements in the grid-cell cognitive space. An element in this grid-cell cognitive space can be represented as follows:

5 $$\begin{aligned} \varvec{\Upsilon _i} = [x^g_i,y^g_i,\varsigma _i] \end{aligned}$$

where $\varsigma _i \in [0,1]$ is the firing rate in the coordinate $\varvec{P^g_i}=(x^g_i,y^g_i)$ of the cognitive space for the grid cell $\varvec{G_j}$. The coordinate $\varvec{P^g_i}$ can be represented by the combination of two basis vectors that span the grid cell cognitive space. The two basis vectors denoted by $\varvec{\epsilon _j^1}$ and $\varvec{\epsilon _j^2}$ in the cognitive space are defined according to the preferred spacing, orientation and phases of grid cells as follows:

6 $$\begin{aligned} \varvec{\epsilon _j^1} = [s_j cos(\theta _j),s_j sin(\theta _j)] \end{aligned}$$

7 $$\begin{aligned} \varvec{\epsilon _j^2} = [s_j cos(\theta _j+\pi /3),s_j sin(\theta _j+\pi /3)] \end{aligned}$$

Then the relationship between the place-cell frame and the grid-cell frame is depicted in Fig. 2. The preferred phases of the grid cell denote the translation of the origin of two frames. The preferred orientation of the grid cell describes the angle between two frames. Without loss of generality, the basis vectors of the place-cell frame $\mathcal {C}$ can be simply defined as the standard basis vectors:

8 $$\begin{aligned} \begin{aligned} \varvec{\kappa _1} = [1,0]\\ \varvec{\kappa _2} = [0,1] \end{aligned} \end{aligned}$$

Then, as mentioned above, we think place cells provide the impact of the external cues for the grid cells. The various place-cell frames are provided by place cells via external cues in our model to exert influence on grid patterns. In addition, some findings show that the grid-like representation may be Euclidean spatial metric (Banino et al. 2018). So the linear transformation between place-cell and grid-cell frames herein is depicted as follows:

9 $$\begin{aligned} \begin{bmatrix} \varvec{\kappa _1},\varvec{\kappa _2} \end{bmatrix} \cdot \begin{bmatrix} x^p_i \\ y^p_i \end{bmatrix} = \begin{bmatrix} \varvec{\epsilon _1},\varvec{\epsilon _2} \end{bmatrix} \cdot (\begin{bmatrix} x^g_i \\ y^g_i \end{bmatrix}+ \begin{bmatrix} \vartheta _j^1 \\ \vartheta _j^2 \end{bmatrix} ) \end{aligned}$$

Then, the coordinate $\varvec{P^g_i}=(x^g_i,y^g_i)$ can be computed as follows:

10 $$\begin{aligned} \begin{bmatrix} x^g_i \\ y^g_i \end{bmatrix} = \begin{bmatrix} \varvec{\epsilon _1},\varvec{\epsilon _2} \end{bmatrix}^{-1} \cdot \begin{bmatrix} \varvec{\kappa _1},\varvec{\kappa _2} \end{bmatrix} \cdot \begin{bmatrix} x^p_i \\ y^p_i \end{bmatrix} - \begin{bmatrix} \vartheta _j^1 \\ \vartheta _j^2 \end{bmatrix} \end{aligned}$$

Substitute Eqs. (6) and (7) into above equation, we can furthermore obtain:

11 $$\begin{aligned} \begin{bmatrix} x^g_i \\ y^g_i \end{bmatrix} = \begin{bmatrix} s_i cos(\theta _i)&{} s_i cos(\theta _i+\pi /3)\\ s_i sin(\theta _i)&{} s_i sin(\theta _i+\pi /3) \end{bmatrix}^{-1} \begin{bmatrix} x^p_i \\ y^p_i \end{bmatrix} - \begin{bmatrix} \vartheta _j^1 \\ \vartheta _j^2 \end{bmatrix} \end{aligned}$$

We can reformulate the above equation in a simplified form as follows:

12 $$\begin{aligned} \varvec{P^g_i}&= \varvec{R_{pg}}\cdot \varvec{P^p_i} - \varvec{\vartheta _j} \end{aligned}$$

where

13 $$\begin{aligned} \varvec{R_{pg}} = \begin{bmatrix} s_i cos(\theta _i)&{} s_i cos(\theta _i+\pi /3)\\ s_i sin(\theta _i)&{} s_i sin(\theta _i+\pi /3) \end{bmatrix}^{-1} \end{aligned}$$

Furthermore, it can be described as a more compact form as follows:

14 $$\begin{aligned} \varvec{P_i^{g\dagger }}= \varvec{T_{pg}}\cdot \varvec{P_i^{p\dagger }} \end{aligned}$$

Here

15 $$\begin{aligned} \varvec{T_{pg}} = \begin{bmatrix} \varvec{R_{pg}} &{} \varvec{p_j}\\ 0 &{} 1 \end{bmatrix},\varvec{P_i^{g\dagger }} = [x^g_i,y^g_i,1]^T \end{aligned}$$

By this, given a position in place-cell frames, the position can be transformed into the grid-cell cognitive space. The firing rate $\varsigma _i$ is calculated by the distance between the stimulation position and the preferred position of the grid cell. For a position $\varvec{P^p_i}$ in the world frame, its corresponding point $\varvec{P^g_i}$ in the firing space can be calculated by Eq. (14). Then its firing rate can be computed as follows:

16 $$\begin{aligned} \varsigma _i = arctan\left(\kappa (\frac{d_i}{s_j}-\zeta )\right) \end{aligned}$$

where $\zeta$ is the threshold of firing, $\kappa$ is a parameter to adjust the size of the bump, $s_j$ is the spacing of the grid cell and $d_i$ is the distance between the stimulation position and preferred position of the grid cell. The preferred position is a point where every element of the coordinate is an integer in the grid-cell frames. So $d_i$ can be calculated as follows:

17 $$\begin{aligned} d_i = (\varvec{P^g_i}-[\varvec{P^g_i}])^T(\varvec{P^g_i}-[\varvec{P^g_i}]) \end{aligned}$$

where $[\cdot ]$ represents a round function, which can convert decimals into integers.

When the rat moves a distance and arrives at a new environment, the external cues of the current position become different from the previous position. A new place cell is stimulated by external cues in the new environment. For this reason, the current place-cell local frame $\mathcal {C}_2$ in the cognitive space will be formed. As Fig. 2 depicted, the grid-cell frame correspondingly is altered due to the relationship between the place-cell frame and the grid-cell frame. According to Eq. 14, the firing field of the grid cell based on the new place-cell frame $\mathcal {C}_2$ emerges. The evidence suggests that if the current environment is connected with the previous environment, the rat will gradually realize the condition with the increasing experience (Carpenter et al. 2015). The firing field of the grid cell can remap to form a global coherent representation. Without loss of generality, the initial place-cell frame $\mathcal {C}_1$ is regarded as a global frame. In our model, to acquire a global coherent representation, the place-cell frame $\mathcal {C}_2$ only needs to be transformed into the global frame, i.e., $\mathcal {C}_1$. Some results support the idea that spatial signals contain egocentric information and allocentric representation in the hippocampal system and they can be transformed reciprocally (Wang et al. 2020; Bicanski and Burgess 2018). This is consistent with the assumption about different place-cell local frames in our model. We analyze the transformation process of two place-cell local frames as follows. Here, the rotation angle and translation vector between the previous place-cell frame $\mathcal {C}_1$ and the current place-cell frame $\mathcal {C}_2$ are defined as $\phi$ and $\varvec{\varpi }$ respectively. Then, for a position $\varvec{P^{p_2}_i}$ in the $\mathcal {C}_2$, its corresponding position $\varvec{P^{p_1}_i}=(x^{p_1}_i,y^{p_1}_i)$ in the global frame can be obtained by transformation as follows:

18 $$\begin{aligned} \varvec{P^{p_1}_i} = \varvec{R_{{p_1}{p_2}}}\cdot \varvec{P^{p_2}_i} + \varvec{\varpi } \end{aligned}$$

where $\varvec{R_{{p_1}{p_2}}}$ is the rotation matrix between $\mathcal {C}_1$ and $\mathcal {C}_2$, which can be described by the rotation angle $\phi$ as follows:

19 $$\begin{aligned} \varvec{R_{{p_1}{p_2}}} = \begin{bmatrix} cos\phi &{} -sin\phi \\ sin\phi &{} cos\phi \end{bmatrix}^{-1} \end{aligned}$$

For simplicity, the $\varvec{P^{p_1}_i}=(x^{p_1}_i,y^{p_1}_i)$ in current place-cell frame can be computed as follows:

20 $$\begin{aligned} \varvec{P_i^{{p_1}\dagger }} = \varvec{T_{{p_1}{p_2}}}\cdot \varvec{P_i^{{p_2}\dagger }} \end{aligned}$$

where $\varvec{P_i^{{p_2}\dagger }}= [x^{p_1}_i,y^{p_1}_i,1]^T,\varvec{P_i^{{p_2}\dagger }}= [x^p_i,y^p_i,1]^T$ and $\varvec{T_{{p_1}{p_2}}}$ represents the transformation matrix, which is composed by $\varvec{R_{{p_1}{p_2}}}$ and $\varvec{\varpi _{{p_1}{p_2}}}$ as follows:

21 $$\begin{aligned} \varvec{T_{{p_1}{p_2}}} = \begin{bmatrix} \varvec{R_{{p_1}{p_2}}} &{} \varvec{\varpi _{{p_1}{p_2}}}\\ 0 &{} 1 \end{bmatrix} \end{aligned}$$

In this paper, the $\varvec{T_{{p_1}{p_2}}}$ and $\varvec{\varpi _{{p_1}{p_2}}}$ are used to describe the transformation between different place-cell frames.

### The firing activity of single grid cell

To investigate the firing activity of the gird cell based on our model, a virtual animal path is simulated by randomly walking in a virtual environment. The experiments are separately performed in different environments, including a square environment of size $5\,\textrm{m} \times 5\,$ m and a circular environment with a radius of $2\,$ m. Without loss of generality, in the two environments, the origin points of the world frame and the place cell frame are assumed to be identical, i.e., (0, 0). The grid cell that is used to generate the firing field can be represented as follows:

22 $$\begin{aligned} \varvec{G} = [1.0,\pi /4,0.5,0] \end{aligned}$$

**Fig. 3:**
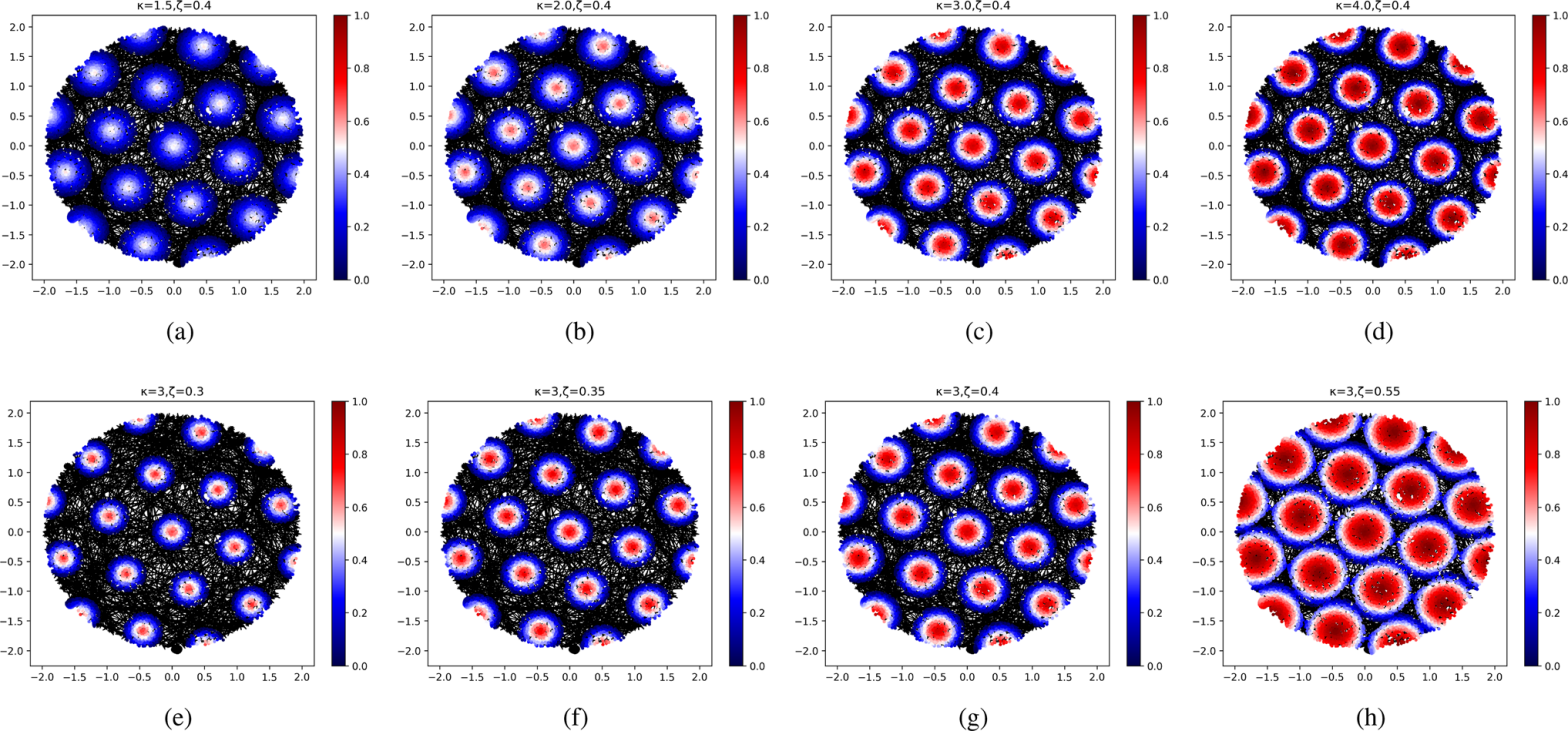
The performance of the model at different $\kappa$ and $\zeta$. $\kappa$ controls the intensities of the firing activities. Greater $\kappa$ gives more intensive activities around the firing center. $\zeta$ controls the range of the firing activities. Greater $\zeta$ gives wider firing fields

The hyperparameters in our model are $\zeta$ and $\kappa$. $\kappa$ controls the intensities of the firing activities. Greater $\kappa$ gives more intensive activities around the firing center, as shown in Fig. 3. $\zeta$ controls the range of the firing activities. Greater $\zeta$ gives wider firing fields, as shown in Fig. 3. To be compatible with the neurophysiological experiments in Hafting et al. (2005), we manually set $\zeta = 0.4$ and $\kappa = 3.0$.

**Fig. 4:**
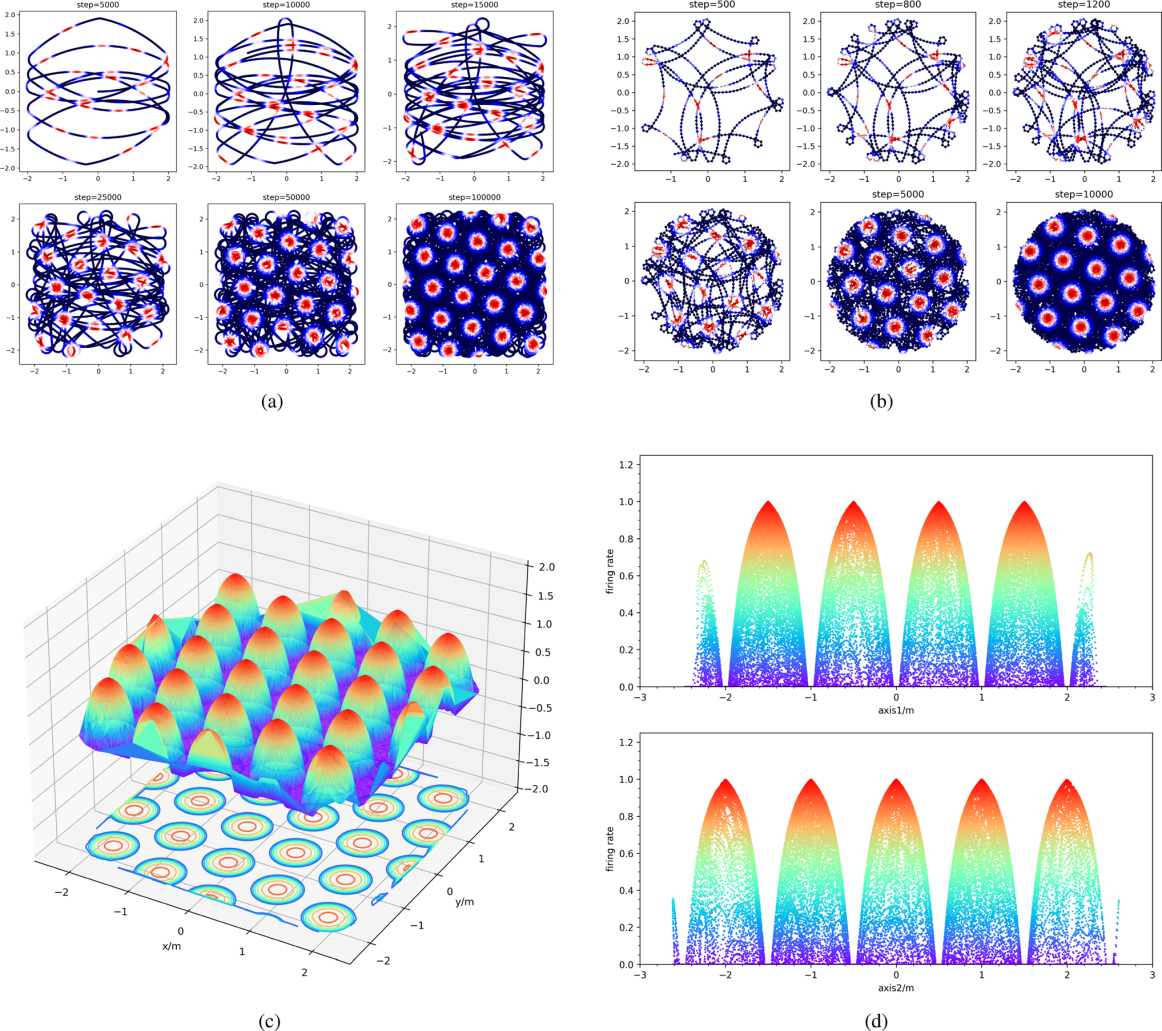
The results of the single grid cell model. **a** The firing activity in a square environment. The black lines represent the trajectories, and different colors including red and blue describe the firing activity of the grid cell. With the movement of the robot, the hexagonal firing field increasingly emerges. **b** The firing activity in a circle environment. **c** The firing rate in a square environment. The 3D surface shows the firing rate in a hexagonal firing field. In the center of the grid node, the firing rate reaches a maximum. **d** The firing rate in a square environment along with two axes. The two axes are the basis vector of the grid cell firing space mentioned above and their phase is (0, 0) to show the phase of the other grid cell. Here, the bumps in the top subfigure are translated by 0.5 m relative to the original point because the phase of the grid cell is [0.5, 0]

In the beginning, the robot is set to the origin point. Due to the monotonous environment, there only is a place-cell frame that is coincident with the global frame. When the robot begins to move, the position $\varvec{P^p_i}$ of every step can be calculated by the speed and the head direction of movement. Then according to Eq. (14), the $\varvec{P^g_i}$ in grid cell firing space can be computed. Finally, the firing rate $\varsigma _i$ of $\varvec{P^g_i}$ can be acquired by Eq. (16). In such a way, every point $\varvec{P^w_i}$ of the place-cell frame in the physical world can be connected with an element in the grid-cell frame.

The firing rates of the grid cell for each point in the trajectories confined in the squared environment are visualized in Fig. 4a. Different firing rates are marked in different colors. The firing rates are ranging from 0 to 1, marked from dark blue to bright red. As Fig. 4a depicted, in the initial period, the firing patterns are inconspicuous because of the limited number of points in the physical world. However, along with the movement, the firing field increasingly begins to emerge the hexagonal firing patterns. This suggests that our grid cell model can generate grid-like patterns. Similarly, the grid-like firing patterns were also observed in the experiment in the circular environment, see Fig. 4b. This demonstrates our model can generate hexagonal firing patterns in different environments.

To further show the firing activities in detail, we visualize the firing rates in the squared environment in 3D as shown in Fig. 4c. The height and size of the bumps in the figure represent the intensity and scope of firing activity. The figure clearly shows the hexagonal firing patterns of the grid cell by our model. The firing rates along the directions of two basis vectors in the grid cell frame are extracted and visualized in Fig. 4d. Through this, we can clearly see the phases of the grid cell. The top subfigure of Fig. 4d shows that the distance between the origin and its closest bump is 0.5. The bottom subfigure shows that the distance between the origin and its closest bump is 0. This is consistent with our settings on the phase of the grid cell in the Eq. (22), i.e., [0.5, 0]. In addition, the spacing of the firing pattern can be easily obtained from Fig. 4d. Obviously, the spacing of the firing pattern is 1.0, which is consistent with our settings in Eq. (22). This demonstrates our model can code the position in the physical world according to the properties of the grid cell.

### The firing activity of multiple gird cells

**Fig. 5:**
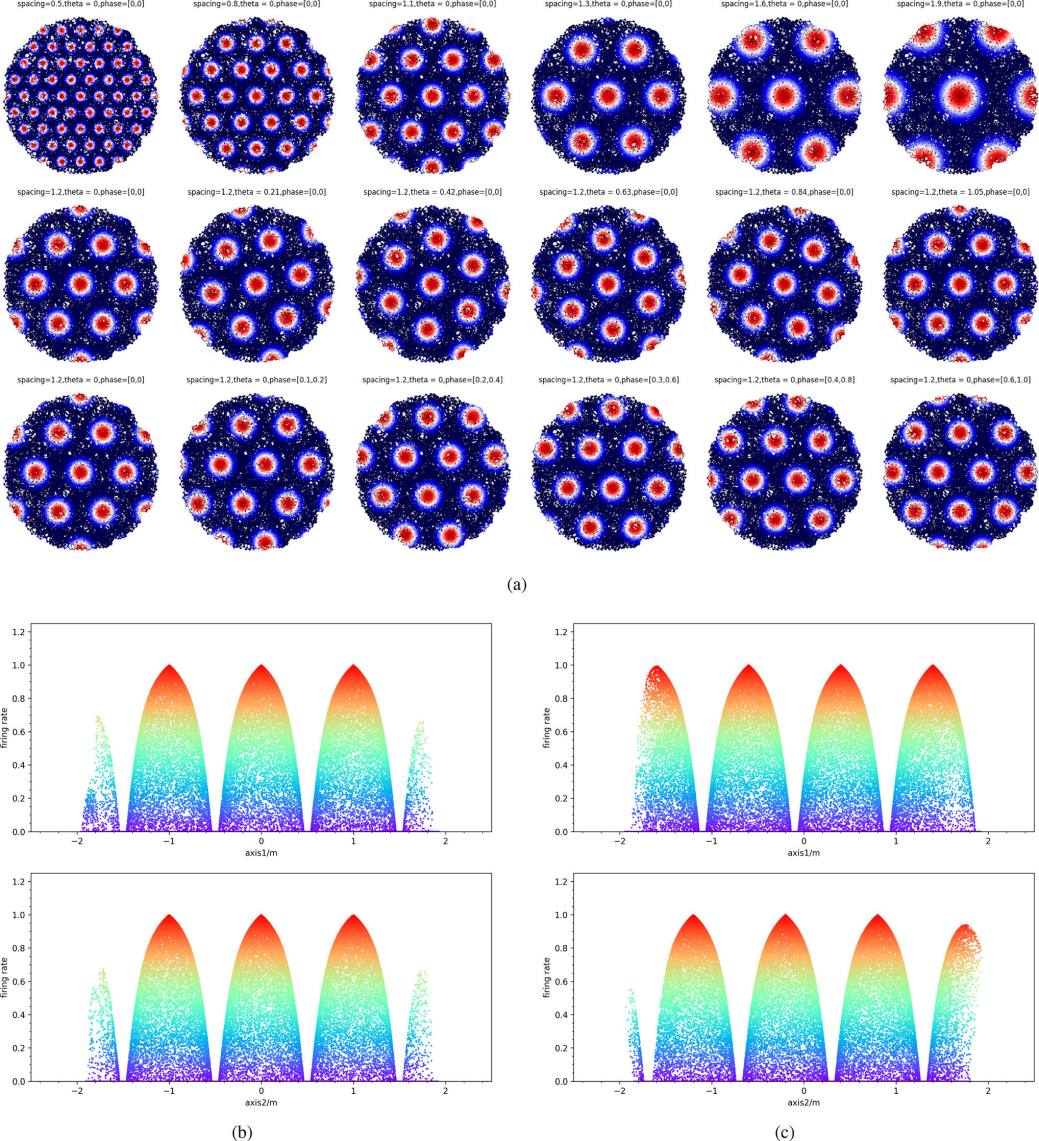
The firing activities of different grid cells. **a** The hexagonal firing pattern of different spacing, orientations and phases. The first line figures show the results of various spacing but the same orientations and phases. The initial spacing is set at 0.5 and it adds 0.3 for the next figure. The figures in the second line demonstrate the results of various orientations and their spacing and phases all set at 1.2 and [0,0]. The figures in the third line show the performance of different phases. **b** and **c** respectively set the phase to [0, 0] and [0.4, 0.8] and show their variety in phases by plotting the firing rate along with two axes

To further explore the performance of our model, we experimented with different grid cells using the circle environment utilized in Sect. 2.2. Other parameters in the Eq. (16) are the same as the Sect. 2.2. For multiple grid cells, the firing activities are shown in Fig. 5. Here, the spacing, phase, and orientation are the characteristics of the grid cell. The changes in spacing, orientation and phases in our model are used to show the firing patterns of different grid cells. The parameters including spacing, orientation and phases of grid cells are manually set and are not trained. Firstly, the grid cells with different spacing are used to generate the firing field. As shown in the top line of Fig. 5a, the intervals between two bumps increasingly expand with the increasing spacing of grid cells. Furthermore, when the spacing of grid cells increases, the size of bumps grows as well. Altering the orientation of the gird cell can also change the angle of firing patterns, see pictures in the middle line of Fig. 5a. Finally, the results of various phases are revealed in the bottom line of Fig. 5a. With the change of phases, the firing patterns begin to translate in the plane. To obviously show the results of various phases, the firing activities along with the two base vectors of the grid-cell frames are shown in Fig. 5b, c. They clearly show the firing activities of two different phases of grid cells. In fact, the phases of the two grid cell are [0, 0] and [0.4, 0.8], respectively.

The above experimental results reveal that our model has the ability to cover the three properties of grid cells including spacing, orientation and phases.

### The global representation in connected environment

**Fig. 6:**
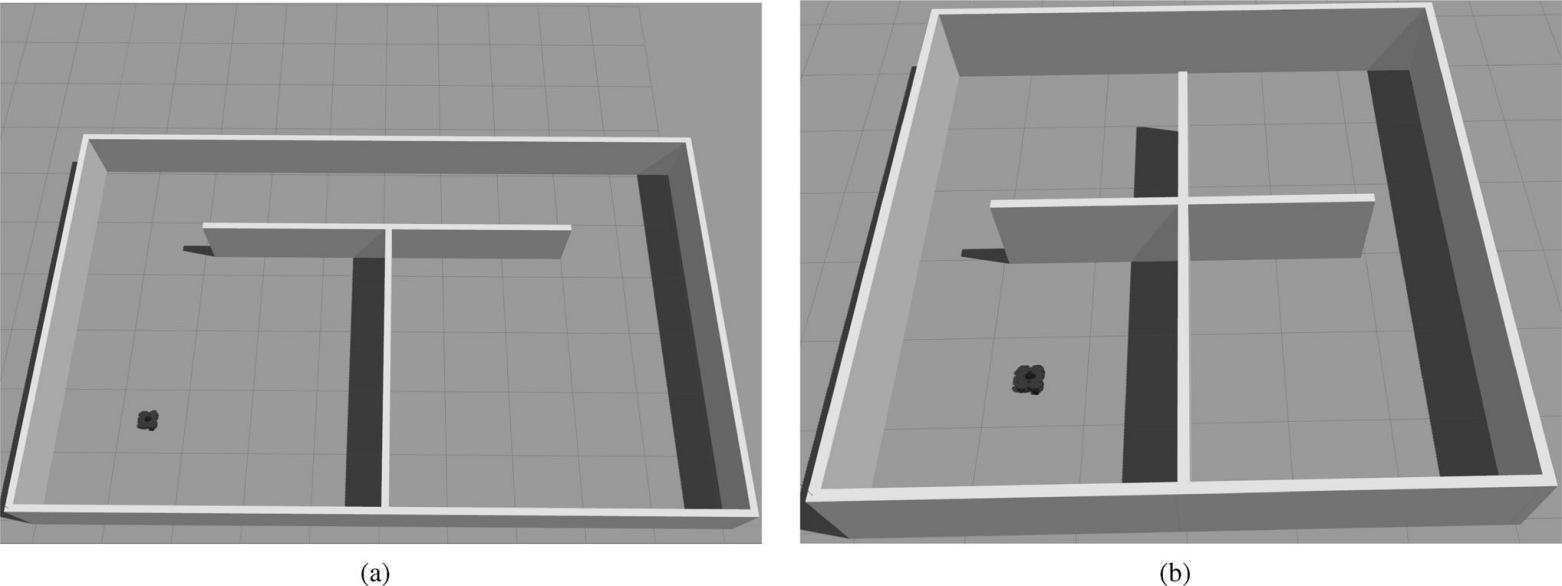
The simulation environments. A robot is regarded as a rodent animal to walk randomly in these environments. In the MEC, the robot’s velocities and directions are used as input for grid cells from speed cells and head direction cells. They are recorded here as input for our model, which generates grid-cell firing patterns. Furthermore, the true trajectories of the robot are reserved to show the grid-cell firing patterns. **a** The environment includes two compartments. The two compartments are connected via a corridor, and they have identical visual cues and sizes. The size of every compartment is $5\times 4$. **b** The environment contains four compartments. The four compartments have the same visual features and sizes. The size of every compartment is $3\times 3$

In the reference (Carpenter et al. 2015), the researchers point out that the grid cells can form a global representation of connected environments. It hypothesizes that grid cell firing activities are determined by external cues or different absolute positions in space. To verify this assumption, they designed an environment containing two perceptually identical compartments connected via a corridor. It is almost the same as the environment that we modeled in Fig. 6a. Their experiments show that grid patterns are initially incoherent in two compartments. However, with increasing experience, discontinuities in grid cell firing patterns in two compartments were incrementally decreased to form a single, continuous firing field that spanned the whole environment. These results show that grid cells can adjust the firing field to generate a globally coherent representation. Their explanation for this phenomenon is that local or global reference frames may determine the grid-cell firing activities.

As mentioned above, the experiment and hypothesis are consistent with our model. Different from the hypothesis, the reference frames herein are place-cell frames and grid-cell frames. According to the conclusion above, a grid cell has a unique grid-cell frame because of its exclusive properties including spacing, orientation and phases. So different place-cell frames are the reason for incoherent firing patterns from the two compartments. In our model, the place-cell frames are regarded as the local frames, which describe the relevance to different environments. When the rodent animal first enters an environment with two compartments in Fig. 6a, the place-cell frames in the two compartments have the same orientation but different origins because of the same visual cues. So according to our model, the grid-cell firing patterns will be identical and only in a different position. With prolonged experience, the rodent animal begins to realize the two compartments are connected via a corridor. In other words, two compartments can be regarded as a connected environment. In a connected environment, the rodent animal only needs a place cell to represent the environment, i.e., a place-cell frame in our model. So another place-cell frame in the right compartments will be adjusted to the global frame according to the relative position relationship of the two compartments. This makes the firing patterns of grid cells increasingly form a coherent global representation.

To explain this process in detail, the environment that is shown in Fig. 6a is used as an example. We assume the place-cell frame in the left compartment is defined as $\mathcal {C}_1$ and that in the right compartment is defined as $\mathcal {C}_2$. When the rodent animal begins to explore the environment, the two place-cell frames in two compartments keep the same orientation relative to the global frame $\mathcal {W}$ because of the fully identical external visual cues. In other words, the difference between $\mathcal {C}_1$ and $\mathcal {C}_2$ only comes from different origin points. In our model, similar to Eq. (21), the relationship between different place-cell frames and the global frame $\mathcal {W}$ can be described using $\varvec{R_{wp_i}}$ and $\varvec{\varpi _{wp_i}}$. So the relationship between two place-cell can be depicted as follows:

23 $$\begin{aligned} \begin{aligned} \varvec{R_{wp_1}}&= \varvec{R_{wp_2}}\\ \varvec{\varpi _{wp_2}}&\ne \varvec{\varpi _{wp_2}} \end{aligned} \end{aligned}$$

Without loss of generality, we can find separately two points $\varvec{P_1^p}$ and $\varvec{P_2^p}$ in place-cell frames $\mathcal {C}_1$ and $\mathcal {C}_2$. In addition, the coordinate of $\varvec{P_1^p}$ is set to be equal to that of $\varvec{P_2^p}$ to represent the same position in two compartments. Then according to Eq. (14), the grid-cell firing fields in $\varvec{P_1^p}$ and $\varvec{P_2^p}$ can be separately calculated as follows:

24 $$\begin{aligned} \begin{aligned} \varvec{P^g_1} = \varvec{T_{pg}} \varvec{P_1^p}\\ \varvec{P^g_2} = \varvec{T_{pg}} \varvec{P_2^p} \end{aligned} \end{aligned}$$

Obviously, we can draw the conclusion that $\varvec{P^g_1}$ and $\varvec{P^g_2}$ are the same. Furthermore, the position $\varvec{P_1^w}$ and $\varvec{P_2^w}$ in global frame corresponding to $\varvec{P_1^p}$ and $\varvec{P_2^p}$ can be calculated by Eq. (18) as follows:

25 $$\begin{aligned} \varvec{P_1^w}&= \varvec{R_{wp_1}^{-1}}\cdot (\varvec{P_1^p} - \varvec{\varpi _{wp_1}}) \end{aligned}$$

26 $$\begin{aligned} \varvec{P_2^w}&= \varvec{R_{wp_2}^{-1}}\cdot (\varvec{P_2^p} - \varvec{\varpi _{wp_2}}) \end{aligned}$$

Then according to Eq. (23), the relationship between $\varvec{P_1^w}$ and $\varvec{P_2^w}$ can be acquired as follows:

27 $$\begin{aligned} \begin{aligned}&\varvec{P_1^w} - \varvec{P_2^w} \\&= (\varvec{R_{wp_1}^{-1}}\cdot (\varvec{P_1^p} - \varvec{\varpi _{wp_1}})) - (\varvec{R_{wp_2}^{-1}}\cdot (\varvec{P_2^p} - \varvec{\varpi _{wp_2}}))\\&=\varvec{R_{wp_1}^{-1}}(\varvec{\varpi _{wp_2}}-\varvec{\varpi _{wp_1}}) \end{aligned} \end{aligned}$$

By Eq. (27), it shows that the difference between $\varvec{P_1^w}$ and $\varvec{P_2^w}$ only is the translation vectors, i.e., $\varvec{\varpi _{wp_1}}$ and $\varvec{\varpi _{wp_2}}$. So the same firing patterns $\varvec{P_1^g}$ and $\varvec{P_2^g}$ are different in their position. In other words, when the rodent animal begins to explore the environment, grid firing patterns were dominated by local environmental cues, replicating between the two compartments.

With the movement in the environment, the rodent animal realizes the two compartments are connected via a corridor. In addition, the relative relationship between two place-cell frames is incrementally perceived. So in this environment, the grid cell is prone to acquire a globally coherent pattern. Here, if $\mathcal {C}_1$ is looked at as a global frame in the environment in Fig. 6a, the $\mathcal {C}_2$ in the right compartment will be incrementally adjusted to the global frame to acquire a globally coherent pattern. Here, due to the same visual cues, the only translation element $\varvec{\varpi _{wp}}$ needs to be adjusted. For more general conditions, the $\varvec{T_{wp}}$ needs to be adjusted.

In the environment in Fig. 6a, to achieve the process, $\varvec{T_{wp_2}}$ needs to be adjusted with the increasing experience of the environment. To describe conveniently, $\varvec{T_{wp_2}}$ can be regarded as a function of time *t* and it can be represented as $\varvec{T_{wp_2}}(t)$. In consideration of identity of the place-cell frame $\mathcal {C}_1$ and the global frame $\mathcal {W}$, the condition should be met by adjusted as follows:

28 $$\begin{aligned} \lim _{t\rightarrow \infty }\varvec{T_{wp_2}}(t) = \varvec{T_{wp_1}} \end{aligned}$$

where $$\varvec{T_{wp_2}}(t_0) = \varvec{T_{wp_2}}$$.

According to Eq. (21), the $\varvec{T_{wp_2}}$ can be represented as follows:

29 $$\begin{aligned} \varvec{T_{wp_2}} = \begin{bmatrix} \varvec{R_{wp_2}} &{} \varvec{\varpi _2}\\ 0 &{} 1 \end{bmatrix} \end{aligned}$$

The rotation matrix $\varvec{R_{wp_2}}$ can be updated iteratively as follows:

30 $$\begin{aligned} \varvec{R_{wp_2}'} = \varvec{\Delta R_t} \cdot \varvec{R_{wp_2}} \end{aligned}$$

where $\varvec{R_{wp_2}'}$ is the rotation matrix after an update, $\varvec{\Delta R_t}$ is a rotation matrix that is generated by a small update angular in time *t*.

Similarly, the translation $\varvec{\varpi }$ can be updated as follows:

31 $$\begin{aligned} \varvec{\varpi _2}' = \varvec{\varpi _2} - \Delta \varvec{\varpi _t} \end{aligned}$$

where $\varvec{\varpi _2'}$ is the translation after update, $\Delta \varvec{\varpi }$ is the decrement for translation in the time*t*. In this paper, the $\varvec{\Delta R_t}$ can be computed by:

32 $$\begin{aligned} \varvec{\Delta R_t} = \begin{bmatrix} cos\Delta \phi &{} -sin\Delta \phi \\ sin\Delta \phi &{} cos\Delta \phi \end{bmatrix}^{-1} \end{aligned}$$

where $\Delta \phi$ is the rotation angular to adjust, calculated as follows:

33 $$\begin{aligned} \Delta \phi = \phi \cdot \frac{\alpha }{\beta +e^{-\xi (N_t-M)}} \end{aligned}$$

where $\phi$ is the angular difference between $\mathcal {C}_1$ and $\mathcal {C}_1$, $\alpha ,\xi ,\beta , M$ is the hyper-parameters that are used to control the speed of the adjustment, $N_t$ is the steps of robot movement in other place cell frame after time *t* to represent the familiarity. To simplify the notation, we denote the parameter as follows:

34 $$\begin{aligned} \gamma = \frac{\alpha }{\beta +e^{-\xi (N_t-M)}} \end{aligned}$$

Then, the $\Delta \varvec{\varpi }$ can be given by:

35 $$\begin{aligned} \Delta \varvec{\varpi _t} = \gamma \cdot \varvec{\varpi _t} \end{aligned}$$

where $\varvec{\varpi _t}$ is the translation difference between $\mathcal {C}_1$ and $\mathcal {C}_1$.

In this way, the new transformation matrix $\varvec{T_{wp_2}'}$ can be represented as follows:

36 $$\begin{aligned} \varvec{T_{wp_2}'} = \begin{bmatrix} \varvec{R_{wp_2}'} &{} \varvec{\varpi _2'} \\ 0 &{} 1 \end{bmatrix} \end{aligned}$$

Then the firing field can be updated by Eq. (14) using the above new transformation matrix. With the iterations, $\varvec{T_{wp_2}}$ gradually reaches $\varvec{T_{wp_1}}$ and the whole firing field incrementally reforms a global coherent representation.

To verify the proposed theory and reproduce the biological experiment results, we designed two environments that have separately two compartments and four compartments. The two environments are described in Fig. 6a, b. Then a robot is used as a rodent animal to explore randomly in these two environments. The positions of the robot can be acquired by its sensors and the corresponding ones are used to generate firing patterns according to our model. The hyper-parameters of our model in two environments are defined as Table.1.

**Table 1:** Values of parameters

Parameters	Two compartments	Four-compartments
$\zeta$	0.4	0.4
$\kappa$	3.0	3.0
$\alpha$	0.5	0.8
$\beta$	1.0	1.0
$\xi$	0.1	0.2
*M*	100000	100000
*G*	$[1.5,\pi /4,0,0]$	$[1.0,\pi /4,0,0]$

**Fig. 7:**
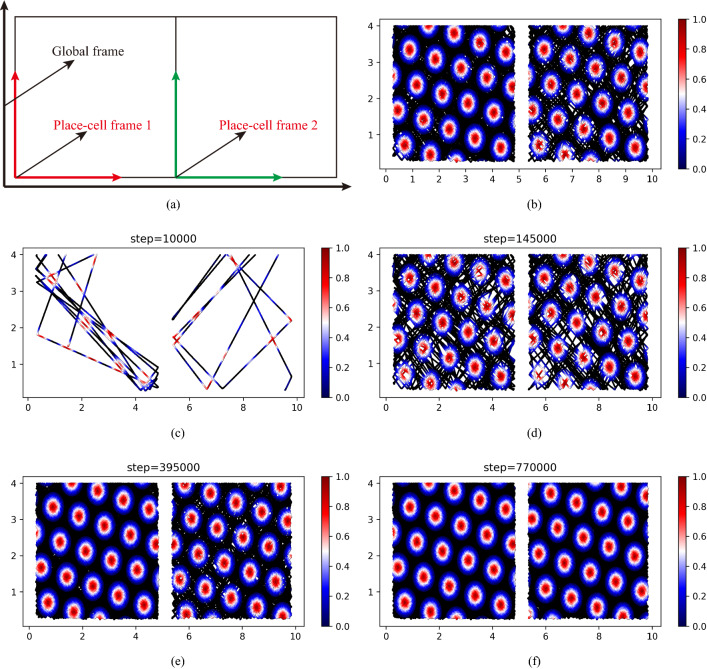
The results in the environment include two compartments. The blank lines in the figures represent the trajectories of the rodent animal. The various colors ranging from red to blue denote values of the firing rates of the grid cell. **a** The place-cell frames and global frames in the experiment. **b** The firing patterns of the grid cells without adjustment. Here, the firing patterns in the two compartments are identical because of the same visual cues. **c** The grid-cell firing pattern and trajectories of the rodent animal at 10000 steps. **d** The grid-cell firing pattern and trajectories of the rodent animal at 145000 steps. The hexagonal firing patterns of the grid cell begin to appear. **e** The grid-cell firing pattern and trajectories of the rodent animal at 395000 steps. The firing patterns have gradually been reformed by adjusting the place-cell frames. **f** The grid-cell firing pattern and trajectories of the rodent animal at 770000 steps. At this time, the firing patterns have formed a global coherent representation

Firstly, the experiment is performed in the environment including two compartments depicted in Fig. 6a. Here, the origin point of the place-cell frame in every compartment is specified in its left bottom corner. The detail of place-cell frames and the global frame are depicted in Fig. 7a. In addition, their basis vectors are set as the standard basis vectors in Eq. (8). The experiment is first performed without adjustment operations. The results are depicted in Fig. 7b. The black lines in the figure are the trajectories of the rodent animal and different colors ranging from red to blue represent the value of the firing rates. In addition, the patterns in the corridor are ignored to clearly show the difference between the patterns in the two compartments. The figure shows that the grid-cell firing patterns in two compartments are identical when the whole process lacks adjustment. Without the adjustment, the firing patterns of the grid cell are dominated by local frames, i.e., place-cell frames in each compartment. Since the two compartments have the same visual cues, the two local frames share the same orientation but have their own origins.

**Fig. 8:**
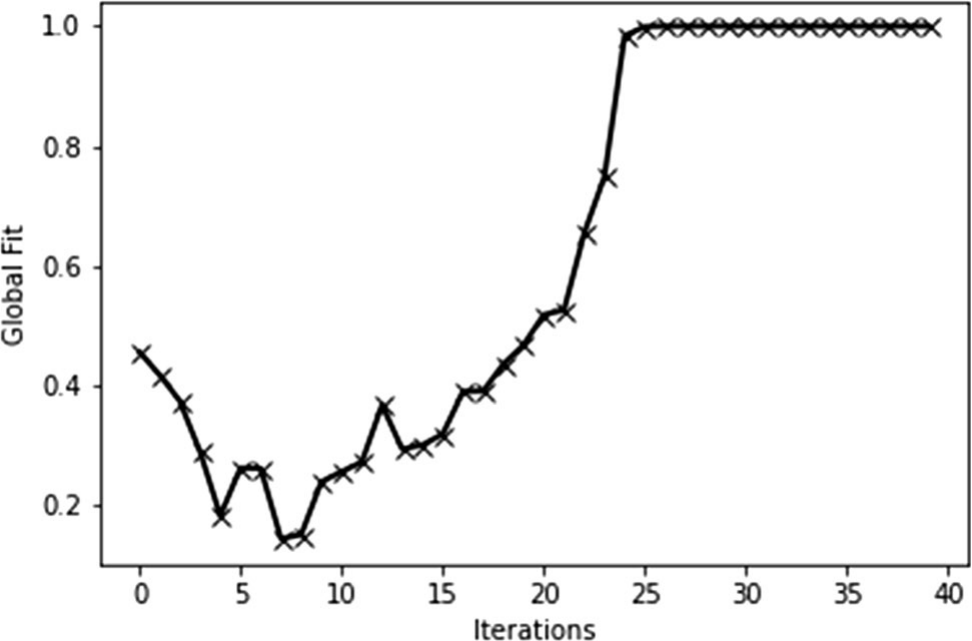
The correlation of global fit in the environment including two compartments

We then performed the experiment with the adjustment operations described above. The experiment results are depicted in Fig. 7. Figure 7c–f represent the change of firing patterns of the grid cell, as the moving steps increase. During the early sessions in Fig. 7c–e, the grid-like firing patterns of the grid cell were gradually present in the environment. In this phase, the firing patterns of the grid cell in two compartments are incoherent. However, with increasing experience, the firing patterns in the right compartments gradually adjusted and finally acquire a global coherent representation of the whole environment. In addition, to describe the extent of global coherent representation, the Pearson correlation coefficient is introduced. Figure 8 shows the correlation of global fit gradually raises with the movement of the rodent animal. This demonstrates whole gird-cell firing patterns gradually reformed a global coherent representation. Here, we reproduce the experiment results about grid-cell global representation in a connected environment. It demonstrates that our model supports the conjecture about the reason for global representation in a connected environment. This also laterally shows that our model may be the internal mechanism of the grid firing field.

**Fig. 9:**
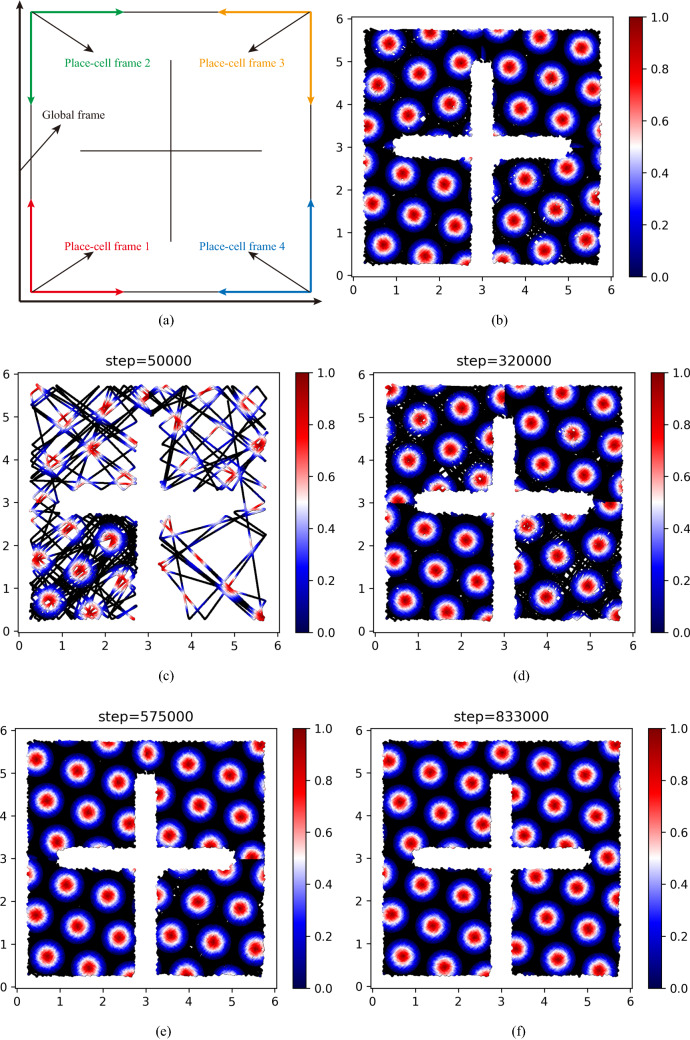
The results in the environment include four compartments. The blank lines in the figures represent the trajectories of the rodent animal. The various colors ranging from red to blue denote values of the firing rates of the grid cell. **a** The place-cell frames and global frames in the experiment. **b** The firing patterns of the grid cells without adjustment. **c** The grid-cell firing pattern and trajectories of the rodent animal at 50000 steps. The hexagonal firing patterns of the grid cell begin to appear. **d** The grid-cell firing pattern and trajectories of the rodent animal at 320000 steps. The firing patterns have gradually been reformed by adjusting the place-cell frames. **e** The grid-cell firing pattern and trajectories of the rodent animal at 575000 steps. The firing patterns of the compartment in the upper left and upper right corners have formed a coherent representation. **f** The grid-cell firing pattern and trajectories of the rodent animal at 833000 steps. At this time, the firing patterns in all compartments have formed a global coherent representation

As mentioned above, the place-cell frames in the two compartments in our experimented environment have the same orientation and only are different in origin points. To give a prediction in a more complex environment, the environment containing four compartments is designed, shown in Fig. 6b. Every compartment in this environment is assigned a place-cell frame when the rodent animal first enters the compartments. These place-cell frames and the global frame are depicted in Fig. 9a. In the figure, different color frames represent different place-cell frames. As the figure depicted, the different place-cell frames not only have different origin points but also have different orientations. So unlike the results in the environment only including two compartments, the firing patterns in different compartments here are various when the process lacks adjustment operations. These results are described in Fig. 9b. In addition, the grid-cell firing patterns in a compartment can be obtained from other firing patterns in other compartments by rotation and translation, as the place-cell frames in these compartments can be transformed to each other by rotation and translation, shown as Fig. 9a.

**Fig. 10:**
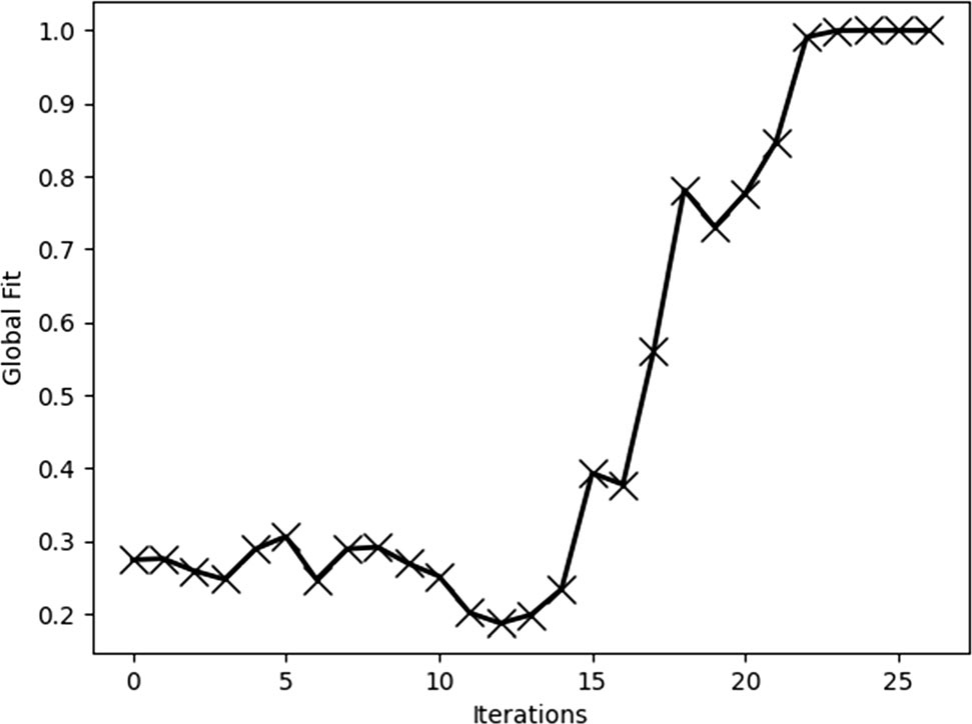
The correlation of global fit in the environment including four compartments

The prediction results of our proposed grid-cell model are depicted in Fig. 9c–f. The figures describe the grid-cell firing patterns under different numbers of moving steps. The black solid lines represent the trajectories of the rodent animal and the different colors ranging from red to blue denote the values of the grid-cell firing rates. In the initial stage, the hexagonal firing patterns of the grid cell gradually begin to emerge. However, as Fig. 9c–e depicted, the firing patterns in different compartments are incoherent and independent. With the increasing experience, these firing patterns in four compartments incrementally adjust and reform a global coherent representation (Fig. 9f). The results demonstrate the reason for incoherent grid-cell firing patterns comes from different place-cell frames in four compartments. When the place-cell frames gradually are adjusted with the increasing experience, the firing patterns of the grid cell will be transformed into the same firing space to form a global coherent representation. Similarly, the correlation of global fit can be depicted in Fig. 10. Figure 10 shows the correlation of global fit gradually raises with the movement of the rodent animal. It demonstrates gird-cell firing patterns gradually formed as a global coherent representation.

## Discussion

The grid cells are an important discovery in the hippocampal-entorhinal system. In moving rodent animals, the hexagonal firing fields of a grid cell can tile the entire available space. The main view thinks that grid cells provide context-independent spatial metrics to represent the local environment. However, more and more evidence demonstrates that grid cells can be affected by external cues (Hafting et al. 2005; Barry et al. 2012; Chen et al. 2016; Pérez-Escobar et al. 2016; Krupic et al. 2015). So the relative contribution of self-motion inputs to grid-cell activity might have been overestimated (D’Albis and Kempter 2017). The grid cells can maintain the firing patterns in the dark, which maybe come from olfactory cues or somatosensory inputs. And the firing fields in the dark can maintain stability in rats (Hafting et al. 2005; Barry et al. 2012), but not in mice (Chen et al. 2016; Pérez-Escobar et al. 2016). Carpenter et al. (Carpenter et al. 2015) investigate global representation in the connected environment for grid cells. The rats are allowed to run in an environment connected by two identical corridors. In the beginning, the firing fields in the two corridors are the same but incoherent. With increasing experience, the firing patterns in two corridors will gradually adjust to form a global representation of the entire environment. The authors hypothesize that if grid cells are dominated by external cues, two local and identical patterns could emerge. If self-motion inputs are prevailing, the grid-cell firing field in the entire environment will be a global representation. The results demonstrate that external cues may have a significant influence on grid-cell activity initially and self-motion inputs are prevailing with increasing experience. It demonstrates that grid cells need to receive external cues and self-motion inputs to form the hexagonal firing field. However, the OI models more focus on the self-motion inputs and the single-cell adaptation models emphasize the external cues. Although the CAN models can utilize both self-motion inputs and external cues, the external cues make little contribution to the generation of grid-like patterns. As the CAN models can generate grid-like patterns only in the presence of self-motion inputs. The external cues are only utilized to correct the errors from the self-motion inputs. Different from CAN models, our idea is to tightly couple the self-motion cues and external cues to provide an insight to understand how place cells and grid cells interact with each other.

We propose a novel model from a new insight base on cognitive spatial transformation, which can utilize both external cues and self-motion inputs to generate the hexagonal firing patterns. The core of our model is that the external information in physical space is transformed into grid-cell cognitive space. Consistent with the evidence that the deep entorhinal layer(layers V and VI) receive direct synaptic projections from the hippocampus (Sürmeli et al. 2015; Tamamaki and Nojyo 1995), the external cues in the model are assumed to provide various local frames for grid-cell cognitive space by activating the place cells. In the grid-cell cognitive space, the frame of a grid cell is structured by the preferred spacing, orientation and phases of a grid cell. The self-motion inputs firstly are transformed into local frames according to the activating place cells. After this, the representation in the current environment by place-cell local frame is projected into grid-cell cognitive space according to the corresponding grid cell. The results demonstrate that our model can represent the physical environment in the grid-cell cognitive space. It can fully show three properties of grid cells including spacing, orientation and phases in Fig. 5. Compared with other models, the orientation of grid cells in CAN models (Burak and Fiete 2009; Guanella et al. 2007) is designed artificially and the hexagonal structure in OI models (Burgess et al. 2007) is arranged by 60 degrees. As for the single-cell adaptation models (Kropff and Treves 2008), the orientation of the firing field relies on the head direction of the animal’s movement.

In the Sect. 2.4, our model reproduces the experimental results about the global representation of the connected environment (Carpenter et al. 2015). The authors suggest that the firing fields of grid cells can be affected by external cues in a short time and self-motion inputs in a long time (Carpenter et al. 2015). We herein introduce the place-cell local frames and think they are the key elements for grid-cell firing fields. In case of a short time, the place-cell local frames are determined by external cues. However, with the movement, self-motion inputs will play a more important role in place-cell local frames. By adjusting the place-cell local frames with increasing experience, grid-cell firing patterns in the connected environment can form a global representation. Besides, the prediction in a more complex environment is performed according to our theory. The result demonstrates that our model can reflect the ability to reform a global representation for grid cells. This is consistent with an early research (D’Albis 2018). The research demonstrates that grid patterns can rotate with polarizing visual cues in circular arenas. When the visual cues are rotated manually, the place-cell local frames in cognitive space also are rotated. This makes the whole grid-cell firing patterns reform. In a very similar research (Wernle et al. 2018), the rats are trained in two rectangular compartments A and B separated by a wall. When the firing fields in A and B are finished, the wall is removed and the rat is allowed to explore the entire environment. The grid patterns near the wall will be largely reformed. Conversely, the grid patterns are retained in the distant wall of the box. When environments are merged, grid fields reform quickly in the area where the environments are fused. From our perspective, the external cues in the removed wall change a lot, which makes place-cell local frames largely adjust to meet the new environment. So the grid firing patterns near the removed wall significantly change. In addition, the grid-cell firing patterns only need to be transformed according to new place-cell local frames rather than regenerating the firing patterns. So the grid firing patterns can be rapidly reorganized.

In our model, the place-cell frames are assigned manually in different environments to test our model. The evidence demonstrates that a place cell generally fires in a particular environment (OKeefe 1976). In other words, a place cell can represent an environment. So in our model, an environment corresponds to a place-cell local frame. In our simple environments, the place-cell local frames are placed in the corners of the compartments or in the same position as physical world frames according to significant visual features. However, there is an issue that how the place-cell local frames are placed in more complex environments. How the orientation and origin of the frames should be determined by place cells? This needs more experimental evidence to understand the mechanism. In addition, the proposed model can generate the hexagonally firing patterns of the grid cells. However, it does not implement the metric property of the grid cells yet. In our future work, multiple grid cells with each modeled by the proposed principles established in this paper will be constructed and arranged in the continuous attractor manifold, which has the metric property and path integration ability of the grid cells.

In conclusion, we proposed a new grid-cell model from a new perspective based on cognitive spatial transformation. Our model established a theoretical framework of the interaction between place cells and grid cells for encoding and transforming positions between the local frame and global frame. The place cells provide various place-cell local frames to affect the grid-cell patterns via external cues. The self-motion inputs are transformed into grid-cell cognitive space by these place-cell local frames and generate grid-like patterns. The grid-cell cognitive space is determined by the preferred spacing, orientation and phases of grid cells. The model not only reproduces the experiment results (Carpenter et al. 2015), but also predicts the results in a novel connected environment. It indicates that the reformation of grid patterns may raise by transforming generated patterns rather than regenerating new patterns. This provides a new insight to understand how the place cells and grid cells integrate external and self-motion cues.

## Materials and methods

A robot is regarded as an animal to explore the environment. The animal can acquire its position in the environment by its velocity and angular. Here, we build three virtual environments including square environment(Fig. 6), two-compartment environment(Fig. 6a), four-compartment environment(Fig. 6b). These virtual environments are customized using open-source modeling software called Blender (https://www.blender.org) and the walls of these environments are set to 0.1 m in thickness and 0.5 m in height.

To validate the proposed model of grid cells, simulations of realistic environments were performed in Gazebo (https://gazebosim.org). A rodent-like mobile robot called Turtlebot3 waffle Pi(https://www.robotis.us/turtlebot-3/) is used to explore the environment. The positions of the robot in environments can be acquired by its sensors.

The python language is used here to implement the model and show results. The robot and simulation environment is implemented using C++ language, which is performed in Robot Operating System (ROS, https://www.ros.org/) neotic on Ubuntu 20.04 LTS.
